# Integrating peptides' sequence and energy of contact residues information improves prediction of peptide and HLA-I binding with unknown alleles

**DOI:** 10.1186/1471-2105-14-S8-S1

**Published:** 2013-05-09

**Authors:** Fei Luo, Yangyang Gao, Yongqiong Zhu, Juan Liu

**Affiliations:** 1 School of Computer, Wuhan University, Wuhan, Hubei, China

## Abstract

**Background:**

The HLA (human leukocyte antigen) class I is a kind of molecule encoded by a large family of genes and is characteristic of high polymorphism. Now the number of the registered HLA-I molecules has exceeded 3000. Slight differences in the amino acid sequences of HLAs would make them bind to different sets of peptides. In the past decades, although many methods have been proposed to predict the binding between peptides and HLA-I molecules and achieved good performance, most experimental data used by them is limited to the HLAs with a small number of alleles. Thus they are inclined to obtain high prediction accuracy only for data with similar alleles. Because the peptides and HLAs together determine the binding, it's necessary to consider their contribution meanwhile.

**Results:**

By taking into account the features of the peptides sequence and the energy of contact residues, in this paper a method based on the artificial neural network is proposed to predict the binding of peptides and HLA-I even when the HLAs' potential alleles are unknown. Two experiments in the allele-specific and super-type cases are performed respectively to validate our method. In the first case, we collect 14 HLA-A and 14 HLA-B molecules on Bjoern Peters dataset, and compare our method with the ARB, SMM, NetMHC and other 16 online methods. Our method gets the best average AUC (Area under the ROC) value as 0.909. In the second one, we use leave one out cross validation on MHC-peptide binding data that has different alleles but shares the common super-type. Compared to gold standard methods like NetMHC and NetMHCpan, our method again achieves the best average AUC value as 0.847.

**Conclusions:**

Our method achieves satisfactory results. Whenever it's tested on the HLA-I with single definite gene or with super-type gene locus, it gets better classification accuracy. Especially, when the training set is small, our method still works better than the other methods in the comparison. Therefore, we could make a conclusion that by combining the peptides' information, HLAs amino acid residues' interaction information and contact energy, our method really could improve prediction of the peptide HLA-I binding even when there aren't the prior experimental dataset for HLAs with various alleles.

## Background

In the cellular immune system, peptide binding to MHC (Major Histocompatibility Complex, in humans MHC is also called Human Leukocyte Antigen HLA) is the most selective step in recognition of pathogens. In humans, there are three types of MHC molecules and their recognizing, binding, transporting and functioning mechanism are distinct. Taking the MHC-I for example. Proteins in the cytosol are first degraded by the proteasome, and then peptides are internalized by TAP (transporter associating with antigen processing) channel in the endoplasmic reticulum, where MHC-I molecules freshly are synthesized. Complexes of MHC-I binding to peptide enter Golgi apparatus and finally externalize on the cell membrane to interact with T lymphocytes. Correctly and precisely predicting the T cell epitope has realistic meaningfulness, especially important for the vaccine design. Many experiments on analyzing the binding complexes of peptide and MHC indicate that the binding sites have the binding specificity. This specificity is usually determined by the molecular weight, the electric charge, the pH value and other attributes. In the past decades, many prediction methods have been proposed to predict the epitope. They could be categorized into the following types. The first one is the motif matching based methods. From the binding complex fragments of peptide and MHC, Rudensky [1] purified and detected the amino acid sequences, from which they proposed three binding motifs I-A^s^, I-A^b^, and I-E^b^. According to these three motifs, Cole [2] successfully predicted MHC epitopes in the Sendai virus M protein. Analogous methods by checking whether peptides have the anchor sites matching with binding sites in the MHC molecules include works [3-5]. This kind of methods is simple and easy to understand but the prediction accuracy is not high. The second one is the scoring matrix based method. It could be viewed as the generalisation of the motif matching based method. For each type of MHC molecules, the existing peptide-MHC binding data are statistically analysed to generate a coefficient matrix, in which the element represents the degree of amino acid contributing to the binding when appearing in a certain position. Parker [6] used 154 synthetic peptides to get the molecule HLA-A2 scoring matrix; Kubo [7] got the HLA-A1, HLA-A3 and HLA-A11 scoring matrix and Udaka [8] got H2-K^b^, D^b ^and L^b ^scoring matrix respectively. Other similar methods to build up MHC I class and class II molecules' scoring matrix include ProPred [9], ARB [10], SMM [11]. Given the scoring matrix of one MHC molecule, the strength of any peptide binding to it could be calculated in an addition or a multiple way. In comparison with the first type method, they usually have higher accuracy, but there still exist some shortcomings like that they assume each amino acid residue to independently affect the binding and ignore the interactions among the amino acid residues. In order to further improve the prediction accuracy, some methods try to consider the whole peptide sequence and establish more complex models to reflect the real situation. This category of methods includes the Bayesian method (Bayesian) [12], HMM (Hidden Markov Model) [13], SVM (Support Vector Machine) [14], ANN (Artificial Neural Network) [15] and so on [16-21]. Before the prediction, the training process is essential for the prediction model. According to the different types of training data used, previous stated methods also could be categorized into the sequence-based methods and the structure-based methods. They have their own advantages and limitations. On one side, the sequence-based methods always adopt machine-learning approaches that need large amount of training data. When the training data is sufficient, they could get good prediction accuracy. In fact, HLA-I is extremely polymorphic. For example, in the database IMGT/HLA (international ImMunoGeneTics project) [22] the number of registered HLA-I (HLA-I has three major gene locus HLA-A, HLA-B and HLA-C.) molecules has exceeded 3000 and that for HLA-II is over 1100. Unfortunately, for most of HLA molecules with different alleles, there are few or even no experimentally obtained binding complex data to facilitate analysing their binding characteristics. Even for those HLAs that have experimental data, by 2010, IMGT/HLA has deposited 893 allele sequences of the HLA-A loci and 1534 allele sequences of the HLA-B loci, which implies the wide existence of polymorphism. Based on one frequently used dataset composed of 35 HLA-I molecules, the binding prediction accuracy of the HLA-I and peptide is reported to reach average 0.9 AUC (Area Under roc Curve), but this dataset is too special and only accounts for a small part of the known HLA-I molecules. Those HLAs with slightly different amino acid sequences may have their own binding specificity to different sets of peptides. Thus, these sequence-based methods are biased towards the known alleles if they use special training data and may have the over-fitting prediction problem. On the other hand, structure-based methods could jump over the obstacles of sequence polymorphism and directly take advantage of the MHC molecule complex's 3D structures and use their empirical force fields as the binding specificity to estimate the binding affinity. However, the available 3D structure dataset is insufficient. Now there are only tens of HLA-I molecules' 3D structures resolved, so the accuracy of structure-based methods is restricted by the inadequate number of 3D structures and is usually lower than sequence-based methods.

In order to overcome the existing problems and achieve better prediction accuracy, we still take machine-learning strategy based on the sequencing data. As mentioned above, HLA-I has more polymorphism than HLA-II. So here we focus on the HLA-I and a method based on the artificial neural network is proposed to predict on HLA-I with unknown alleles where there are limited or even no prior experimentally obtained dataset. Different from other sequence-based methods using ANN, our method not only considers peptide sequence information but also HLAs' amino acid residues and the energy of contact residues. With information integration, our method is expected to predict the binding of peptide and HLA-I with high accuracy.

The rest sections of this paper will be organized as followed. In the method part, a method based on artificial neural network will be introduced, in which the HLAs interaction residues extraction and contact energy computation will be described. The experiments on two datasets will be implemented in the results and discussion part. On dataset is on the benchmark dataset and the other one is on the super-type dataset. The performance of our method will be evaluated under these two conditions by comparing with other methods. The last part is the conclusion.

## Methods

**Figure 1:**
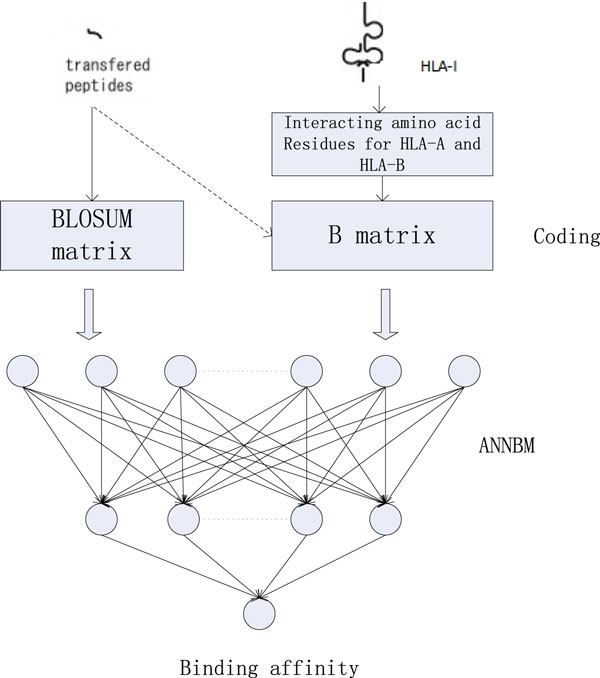
**The framework of our method**. The input data contains two parts, one is the peptide and the other is HLA molecule. HLA molecules will be processed by the steps of extracting interacting amino acid residues and computing the contact energy. Then they will be encoded as the classification features and input into the established classifier to do the training and predict.

There're lots of classifiers could be used for prediction. Choosing a proper classifier relies on the application contexts. Here, we use ANN (Artificial Neural Network) classifier to predict the peptide and HLA-I binding. The reasons for utilizing the ANN model are their advantages of self-learning, self-adaptive and modelling non-linear relationship. Some works [23,24] have proved that ANN is suitable for the epitope prediction. Besides the classifier, how to select the classification features is another important step. Not all of HLA's amino acids take action in the binding process. Therefore, we will first find out which amino acid residues really function and then compute their contact energy. These prominent features will be properly encoded and finally used to predict. The Figure 1 gives the framework of our method.

### Interacting residues of HLA and peptide

**Figure 2:**
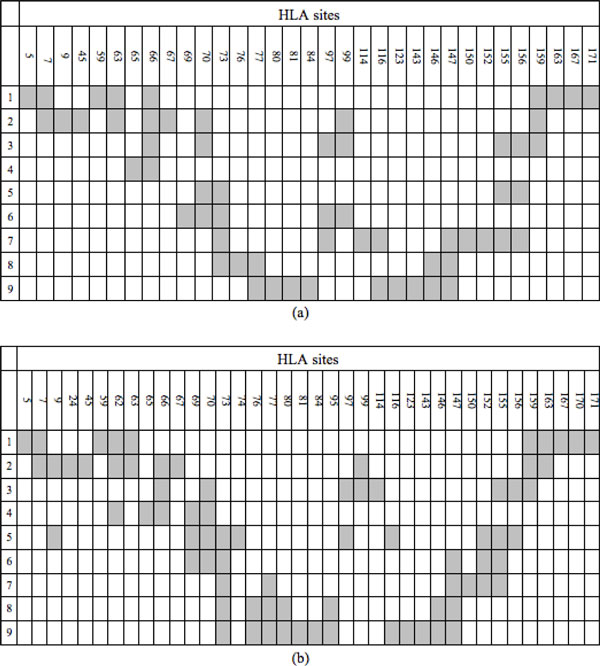
**Interacting residues**. (a) is the binding sites of HLA-A and (b) is the binding sites of HLA-B. The column number represents the HLA molecular residue index given by the IMGT/HLA database and the row number indicates the amino acid residue index of peptide with the length 9. The grey cells in the grid indicate residues that have interaction between HLA and peptide.

The crystal structures of peptide-HLA binding complexes show that although HLA molecules from the same gene locus have different alleles, the complexes have similar spatial structure. Madden [25] have resolved the 3D structures of five complexes where different peptides binding to the same HLA-A*0201 molecule. The results show they have similar structure and HLAs' amino acid residue binding sites. Inspired by Madden's work, one approach to overcome the influence of coding genes' polymorphism is to find out the major common interacting sites of HLAs from the same gene locus. We collect, calculate and finally get the frequent function residues of HLA from the existing structure data of peptide-HLA binding complexes. All raw peptide and HLA structure data come from the database PDB (protein Data Base). In total, there're 111 peptide-HLA-A binding complexes and 87 peptide-HLA-B binding complexes used. When the distance of residues of the HLA and peptide is less than 4 Ȧ, we think that they interact. The results show that for HLAs from the same gene locus, their amino acid residue interacting sites are similar, which is consistent with the result of Madden. We discard those residues of HLAs that interact with peptides less than 5 times. The putative sites are shown in the Figure 2(a) and Figure 2(b) respectively for HLA-A and HLA-B.

### Contact energy of amino acid residues

In fact, as the participants of binding interaction, amino acid residues of HLA and peptide don't independently contribute to the binding. Therefore, it's necessary to take into account the interaction among the amino acid residues. The structure of a peptide-HLA complex is decided by several forces such as the interaction between amino acids and water molecules, the interaction between amino acids and so on. Although there are several forces, the interaction between amino acids plays a major role. Generally, the protein structure could be estimated by the amino acid sequence and the interaction strength could be calculated by the classical electromagnetic theory. If the attracting strength between two amino acid residues is great, their position will have more chance to be near. Therefore, if there are sufficient protein structure data, the mutual attracting strength could be computed. In the structure biology, Miyazawa and Jernigan [26] matrix has been widely used for computing the protein sequence energy in different structure templates. But there's a limitation that Miyazawa and Jernigan matrix takes the solvent as the reference status and is usually accurate for nonpolar molecules interaction. But it couldn't ignore the interaction between amino acids and water molecules. Thus, we use the B matrix [27], which is based on Miyazawa and Jernigan matrix but refers to the threonine, to get the contact energy of amino acid residues.

### Encode the peptide and HLA

For the existing prediction methods based on the artificial neural network, there are three approaches to encode the peptide. The first one is the parse matrix, in which each amino acid is represented by 19 zeros and 1 one. The second one is BLOSUM matrix and the third one is to select some attributes from the physical and chemical properties of amino acids. Among them, BLOSUM matrix usually has the best prediction accuracy and good ability of distinguishing the amino acids. In this paper, the BLOSUM matrix will encode peptides and the B matrix of the contact energy of amino acid residues will encode HLA sequence. Therefore, the encoding length for the HLA-A and peptide binding is 239 dimensions, in which 180 dimensions are for the peptide encoded by BLOSUM matrix and 59 dimensions are for HLA-A amino acid residues interacting with the peptides encoded by the B matrix. The encoding length for the HLA-B and peptide is 255 dimensions, in which 180 dimensions are for peptide encoded by BLOSUM matrix and 75 dimensions are for HLA-B amino acid residues interacting with the peptides encoded by the B matrix. In order to better measure the difference of affinity and facilitate the artificial neural network training. The affinity will be transformed to the logarithm format varying from 0 to 1 like [15].

(1) $$affinity=1-\frac{\text{log}\left(IC50\right)}{\text{log}\left(50000\right)}$$

### Build and train ANNBM

There're several subtypes in the ANN. In this paper we use the error back propagation feed-forward neural network. Because HLA features are encoded by B Matrix, our ANN predictor is also specially named as ANNBM. Theoretical analysis proves that ANN with one single hidden layer can map almost continuous relationship function. Therefore we establish the ANNBM consisting of three layers including an input layer, a hidden layer and an output layer. Neural network's input layer has 239 nodes for predicting peptide-HLA-A binding, while neural network's input layer has 255 nodes for predicting peptide-HLA-B binding. The output layer has only one node. It is the logarithmic value of binding affinity between HLA and peptide.

When constructing the ANNBM, there is no golden criterion to determine the number of nodes in the hidden layer. With less hidden nodes, the ability of learning from samples is poor and unable to reflect the relationship perfectly; with excessive hidden nodes, it may remember the noise in the samples leading to the over learning problem and reduce the generalization ability. In principle, the number of hidden nodes depends on the training sample scale, the sample noise and the complexity of the relationship. A common way to set the number is called trail-and-error, so we test the hidden nodes varying from 2 to 12. The hidden layer with 9 nodes has gotten the minimum mean square error.

In ANNBM, the activation function still used the sigmoid function. For Sigmoid function when the input of variables is very big, its slope trends to 0.

(2) $$f\left(x\right)=\frac{1}{1+e^{-x}}$$

Because of this characteristic, for some learning algorithms like the steepest descent algorithm, as the weights and thresholds are far from its best, the gradient is very small and leads to weights and the thresholds correction is very small, so we use the RPROP method to do network weights adjustment. RPROP takes into account only the sign of the partial derivative over all patterns but not the magnitude, and acts independently on each weight. For each weight, if there is a sign change of the partial derivative of the total error function compared to the last iteration, the update value for that weight is multiplied by a factor η^−^, where η^− ^is less than 1. If the last iteration produces the same sign, the update value is multiplied by a factor of η^+^, where η^+ ^is greater than 1. The update values are calculated for each weight in the manner described as above, and finally each weight is changed by its own update value, in the opposite direction of that weight's partial derivative, so as to minimise the total error function. The parameter η^+ ^is empirically set to 1.2 and η^− ^to 0.5.

## Results and discussion

### Experiment on the benchmark dataset

Bjoern Peters [28] collect peptide-MHC-I binding datasets and builds a benchmark dataset. This benchmark dataset comes from two research groups' works. They are Alessandro Sette in the La Jolla Gene and Immunology Institutes and Søren Buus in the Copenhagen University. Although their experiment systems have several differences in binding the judging index, detecting targets, the preparation of MHC molecules and the purity of peptides, the format of their experiment output is the same. For each peptide, the IC50/EC50 value is assigned to measure its binding affinity. In order to evaluate the consistency of experiment results from these two groups, Bjoern Peters makes the cross validation and results show good consistency. The benchmark finally contains 48 828 binding data on 35 HLA-I molecules. In this paper, we pick up HLA-I with the length 9 to validate our method.

**Figure 3:**
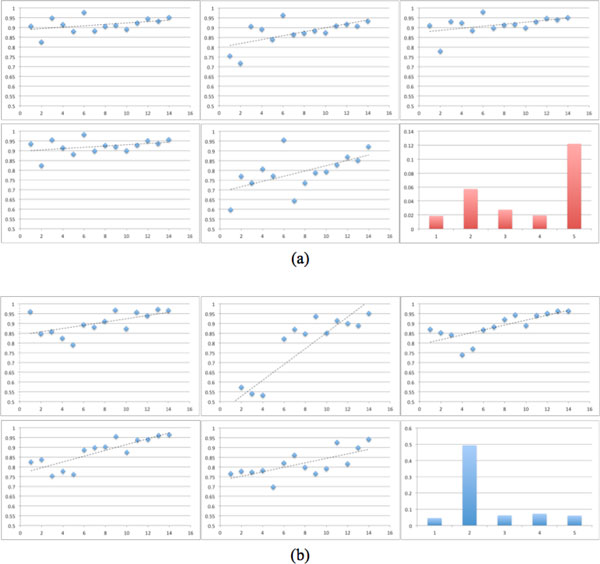
**Methods comparison**. According to the results in the table 1, we divide results into HLA-A class group (a) and HLA-B class group (b) and order them in ascendance based on the peptide number to measure the correlation between scale of dataset and classification accuracy. The panels from left to right and up to down are the linear fitting between the peptide number (x axis) and accuracy (y axis) on five methods: ANNBM, ARB, SMM, NetMHC, and Other methods. The right down picture is the standard deviation of the classification accuracy. We could see ANNBM gets the smallest slope rate and standard deviation, which proves that ANNBM is most independent with dataset scale and stable.

From the table 1, we could see our ANNBM get the best performance. And the prediction is independent with the scale of dataset, which could be observed in the Figure 3. More interesting, the whole prediction accuracy of ANNBM is nearly similar with the second perfect result from the NetMHC and they both belong to the ANN model. Apparently, here the neural networks appear its non-linear mapping advantage that could learn the high order relationship from the training process. Therefore ANN based methods get higher accuracy than SMM and ARB. Besides the model selection, the differences in selecting and encoding of the features also determinate the classification accuracy. SMM and ARB are based on the scoring matrix. NetMHC combines the sparse, BLOSUM matrix and hidden Markov to encode peptides.

**Table 1:** Prediction Results on Benchmark Dataset

Allele	ANNBM	ARB	SMM	NetMHC	Other methods	Peptides
A*0101	0.977	0.964	0.98	0.982	0.955	1157
A*0201	0.951	0.934	0.952	0.957	0.922	3089
A*0202	0.891	0.875	0.899	0.9	0.793	1447
A*0203	0.911	0.884	0.916	0.921	0.788	1443
A*0206	0.906	0.872	0.914	0.927	0.735	1437
A*0301	0.932	0.908	0.94	0.937	0.851	2094
A*1101	0.945	0.918	0.948	0.951	0.869	1985
A*2402	0.826	0.718	0.78	0.825	0.77	197
A*2601	0.950	0.907	0.931	0.956	0.736	672
A*2902	0.907	0.755	0.911	0.935	0.597	160
A*3101	0.923	0.909	0.93	0.928	0.829	1869
A*3301	0.915	0.892	0.925	0.915	0.807	1140
A*6801	0.88	0.84	0.885	0.883	0.772	1141
A*6802	0.883	0.865	0.898	0.899	0.643	1434
B*0702	0.966	0.952	0.964	0.965	0.942	1262
B*0801	0.968	0.936	0.943	0.955	0.766	708
B*1501	0.939	0.9	0.952	0.941	0.816	978
B*1801	0.848	0.573	0.853	0.838	0.779	118
B*2705	0.957	0.915	0.94	0.938	0.926	969
B*3501	0.873	0.851	0.889	0.875	0.792	736
B*4002	0.858	0.541	0.842	0.754	0.775	118
B*4402	0.824	0.533	0.74	0.778	0.783	119
B*4403	0.791	0.461	0.77	0.763	0.698	119
B*5101	0.894	0.822	0.868	0.886	0.82	244
B*5301	0.886	0.871	0.882	0.899	0.861	254
B*5401	0.911	0.847	0.921	0.903	0.799	255
B*5701	0.96	0.428	0.871	0.826	0.767	59
B*5801	0.972	0.889	0.964	0.961	0.899	988

AVG	0.909	0.791	0.874	0.901	0.796	

Table 1 summarizes the comparative results between our ANNBM and the methods in the Bjoern Peters work on the benchmark. We use the AUC (Area Under roc Curve) of 5-folds cross validation as the prediction evaluation criterion. In the table, the first column is the allele name, including 14 HLA-A class molecules and 14 HLA-B class molecules. The columns from 2 to 5 are the AUC value of 5-folds cross validation from the ANNBM、 ARB、 SMM and NetMHC respectively. In addition, ANNBM is also compared to other 16 online prediction methods including various outstanding classifiers like SVM (***arbmatrix, bimas, hlaligand, hla_a2_smm, libscore, mappp, mhcpathway, mhcpred, multipred, netmhc, pepdist, predbalbc, predep, rankpep, svmhc and syfpeithi ***) and the best prediction value among them is listed in the column 6. The last column is the number of peptides binding to the corresponding the HLA molecule.

**Figure 4:**
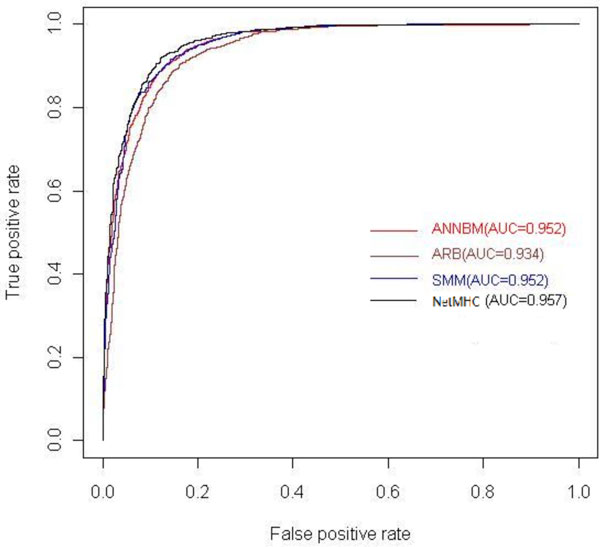
ROC curve of ANNBM、 ARB、 SMM、 NetMHC on HLA-A*0201.

**Figure 5:**
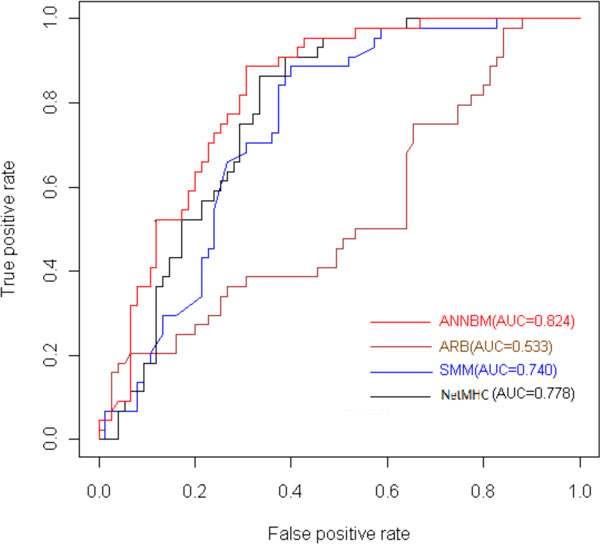
ROC curve of ANNBM、 ARB、 SMM、 NetMHC on HLA-B*4402.

Remarkable, for the small peptide dataset such as B*4002, B*4402, B*4403 and B*5701 molecules, ANNBM outperforms the NetMHC and other methods. The four peptides' numbers are respectively 118, 119, 119 and 59. ANNBM method in the four molecules gets the AUC, respectively, 0.858, 0.824, 0.791 and 0.96, and NetMHC method in the four molecules gets the AUC, respectively, 0.754, 0.778, 0.763 and 0.826. We believe that the ANNBM benefits from taking in account the interaction information of HLA and peptides, whereas NetMHC just encodes the peptides information that is insufficient for ANN to learn the high order relationship under the small datasets situations. Figure 4 shows that when there're enough training dataset the prediction of these methods have no obvious difference just like HLA-A*0201, but under the small dataset situation showed in the Figure 5 ANNBM proves integrating sequence information and energy of contact residues really could improve the prediction accuracy. It isn't hard to understand when training data is sufficient, ordinary classifiers could learn the relationship by large amount of training. The outperformance of ANNBM in the small dataset proves ANNBM effectively catches the key factors for the prediction.

### Prediction on unknown alleles dataset

If there is no prior binding dataset for a HLA molecule, we could make use of the Sette and Sidney work [29] to indirectly solve this obstacle. Sette and Sidney discover that HLA-I class molecules could be divided into several super classes according to their binding specificity. HLA molecules belonging to the same super class have similar binding site structure. Sette and Sidney divide the HLA-A into 5 super classes: A1、 A2、 A3、 A24 and A26,and divide the HLA-B into 7 super classes: B7、 B8、 B27、 B39、 B44、 B58 and B62. In this part, we will validate ANNBM prediction performance when there is no exact binding data for the HLA molecules. Besides the Bjoern Peters' dataset, another 6 HLA molecules are added in from the IEDB [30]. We use the leave-one-out method to do validation. Because the ANN methods have shown better prediction than the other methods on the benchmark dataset, here we only do comparison among three analogous ANN methods. Methods NetMHC and NetMHCpan come from the works [7,31]. NetMHC is the one that has the closest prediction accuracy in the benchmark testing. Here we check whether it could keep performing well. The NetMHCpan is another method designed to predict peptide-HLA binding with unknown alleles. The author of NetMHCpan is an experienced researcher focusing on the MHC epitope prediction. His methods are widely used as the golden standard to evaluate other ones. Finally we get the result shown in table 2, in which the first column is the name of allele, the second column is the super class that the HLA molecule belongs to, columns from third to fifth columns are the AUC value of ANNBM, NetMHC and NetMHCpan and the last column is the number of peptides binding to the corresponding HLA molecule.

**Table 2:** Prediction Results on Unknown Alleles Dataset

Allele	Supertype	ANNBM	NetMHC	NetMHCpan	Peptides
A*0101	A1	0.854	0.672	0.873	1157
A*0201	A2	0.905	0.886	0.912	3089
A*0202	A2	0.840	0.784	0.815	1447
A*0203	A2	0.836	0.818	0.832	1443
A*0206	A2	0.883	0.826	0.847	1436
A*0301	A3	0.867	0.820	0.849	2094
A*1101	A3	0.879	0.851	0.866	1985
A*2301	A24	0.917	0.877	0.863	104
A*2402	A24	0.864	0.848	0.821	197
A*2403	A24	0.923	0.894	0.912	254
A*2601	A1	0.771	0.631	0.733	672
A*2902	A3	0.832	0.603	0.749	160
A*3001	A3	0.863	0.846	0.838	669
A*3002	A1	0.671	0.711	0.721	92
A*3101	A3	0.853	0.822	0.878	1869
A*3301	A3	0.838	0.699	0.763	1140
A*6801	A3	0.768	0.744	0.760	1141
A*6802	A2	0.812	0.664	0.669	1434
A*6901	A2	0.902	0.811	0.823	833
B*0702	B7	0.919	0.864	0.902	1262
B*1501	B62	0.687	0.536	0.750	978
B*1801	B62	0.823	0.775	0.729	969
B*3501	B7	0.805	0.737	0.762	736
B*4001	B44	0.852	0.818	0.870	1078
B*4002	B44	0.883	0.802	0.807	118
B*4402	B44	0.824	0.771	0.839	119
B*4403	B44	0.836	0.800	0.842	119
B*4501	B44	0.822	0.804	0.809	114
B*5101	B7	0.887	0.879	0.905	244
B*5301	B7	0.828	0.819	0.838	254
B*5401	B7	0.880	0.847	0.845	255
B*5701	B58	0.945	0.652	0.919	59
B*5801	B58	0.869	0.625	0.841	988

AVG		0.847	0.774	0.824	

**Figure 6:**
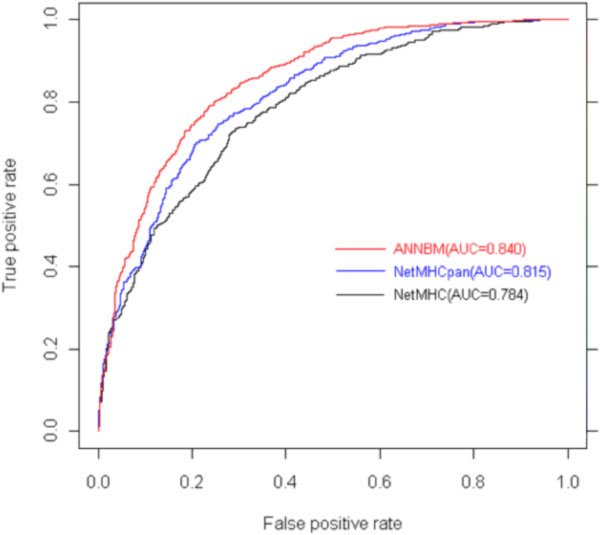
ROC curve of ANNBM, NetMHC and NetMHCpan on A*0202.

**Figure 7:**
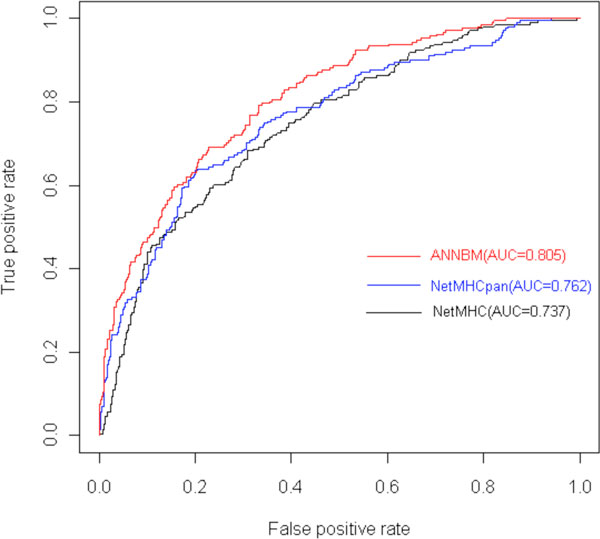
ROC curve of ANNBM, NetMHC and NetMHCpan on B*3501.

From table.2, we can see that ANNBM method obtains the higher average AUC value than NetMHCpan and NetMHC methods by 0.023 and 0.073. NetMHC encoding method doesn't take into account the HLA molecules information. Although the training data comes from the same super-type and acquires perfect results on the allele specific benchmark dataset, the HLA differences in the same super class are not reflected, so it is not difficult to understand the NetMHC prediction accuracy decreases and lower than those of ANNBM and NetMHCpan that encode HLA molecules information. Comparing the encoding method of the HLA molecules between ANNBM and NetMHCpan, ANNBM uses the B matrix and each amino acid that could interact with peptide is denoted by a numerical value, while NetMHCpan uses the BLOSUM matrix and a 20 dimensions vector to denote each amino acid. Obviously, ANNBM has higher efficiency in the storage and computation. The average AUC of ANNBM is greater than that of NetMHCpan, especially on the A*0202 and B*3501, whose ROC curves are showed in figure 6 and 7.

## Conclusions

We have developed an ANNBM method that can well predict peptides binding to HLAs for which have limited or even no prior exact experimentally obtained data. Our method takes both peptide sequence information and contact energy of amino acid residues into account, and could give the quantitative binding affinity. Allele-specific benchmark and super-type experimental datasets successfully validate this method. ANNBM is stably better than other methods, especially for the HLA molecules with the small (< 200) sets of peptides.

## Competing interests

The authors declare that they have no competing interests.

## Authors' contributions

All authors participated in designing and coding the method. FeiLUO drafted the manuscript and all authors read and approved the final version of the manuscript.

## Funding

This work is supported by the grant from the Doctoral Fund of Ministry of Education of China (20110141120031), Central University Scientific Research Fund (211275701), the grants from the National Science Foundation of China (61272274, 60970063), the program for New Century Excellent Talents in Universities (NCET-10-0644), the Ph.D. Programs Foundation of Ministry of Education of China (20090141110026) and the Fundamental Research Funds for the Central Universities (6081007).

## References

[B1] Rudensky A Preston-Hurlburt P al-Ramadi BK Rothbard J Janeway CA Jr Truncation variants of peptides isolated from MHC class II molecules suggest sequence motifs Nature 1992 359 6394 429 431 10.1038/359429a0 1328884

[B2] Cole GA Tao T Hogg TL Ryan KW Woodland DL Binding motifs predict major histocompatibility complex class II-restricted epitopes in the Sendai virus M protein J Virol 1995 69 12 8057 8060 7494321 10.1128/jvi.69.12.8057-8060.1995 PMC189753

[B3] Rammensee H Bachmann J Emmerich NP Bachor OA Stevanovic S SYFPEITHI: database for MHC ligands and peptide motifs Immunogenetics 1999 50 3 213 219 10.1007/s002510050595 10602881

[B4] Doytchinova IA Blythe MJ Flower DR Additive method for the prediction of protein-peptide binding affinity. Application to the MHC class I molecule HLA-A*0201 J Proteome Res 2002 1 3 263 272 10.1021/pr015513z 12645903

[B5] Brusic V Rudy G Harrison LC MHCPEP, a database of MHC-binding peptides: update 1997 Nucleic Acids Res 1998 26 1 368 371 10.1093/nar/26.1.368 9399876 PMC147255

[B6] Parker KC Bednarek MA Hull LK Utz U Cunningham B Zweerink HJ Biddison WE Coligan JE Sequence motifs important for peptide binding to the human MHC class I molecule, HLA-A2 J Immunol 1992 149 11 3580 3587 1331239

[B7] Nielsen M Lundegaard C Worning P Lauemoller SL Lamberth K Buus S Brunak S Lund O Reliable prediction of T-cell epitopes using neural networks with novel sequence representations Protein Sci 2003 12 5 1007 1017 10.1110/ps.0239403 12717023 PMC2323871

[B8] Udaka K Mamitsuka H Nakaseko Y Abe N Empirical evaluation of a dynamic experiment design method for prediction of MHC class I-binding peptides J Immunol 2002 169 10 5744 5753 12421954 10.4049/jimmunol.169.10.5744

[B9] Zhu S Udaka K Sidney J Sette A Aoki-Kinoshita KF Mamitsuka H Improving MHC binding peptide prediction by incorporating binding data of auxiliary MHC molecules Bioinformatics 2006 22 13 1648 1655 10.1093/bioinformatics/btl141 16613909

[B10] Bui HH Sidney J Peters B Sathiamurthy M Sinichi A Purton KA Mothe BR Chisari FV Watkins DI Sette A Automated generation and evaluation of specific MHC binding predictive tools: ARB matrix applications Immunogenetics 2005 57 5 304 314 10.1007/s00251-005-0798-y 15868141

[B11] Peters B Tong W Sidney J Sette A Weng Z Examining the independent binding assumption for binding of peptide epitopes to MHC-I molecules Bioinformatics 2003 19 14 1765 1772 10.1093/bioinformatics/btg247 14512347

[B12] Zhang W Liu J Niu YQ Wang L Hu X A Bayesian regression approach to the prediction of MHC-II binding affinity Computer methods and programs in biomedicine 2008 92 1 1 7 10.1016/j.cmpb.2008.05.002 18562039

[B13] Zhang C Bickis MG Wu FX Kusalik AJ Optimally-connected hidden markov models for predicting MHC-binding peptides Journal of bioinformatics and computational biology 2006 4 5 959 980 10.1142/S0219720006002314 17099936

[B14] Liu W Meng X Xu Q Flower DR Li T Quantitative prediction of mouse class I MHC peptide binding affinity using support vector machine regression (SVR) models BMC bioinformatics 2006 7 182 10.1186/1471-2105-7-182 16579851 PMC1513606

[B15] Buus S Lauemoller SL Worning P Kesmir C Frimurer T Corbet S Fomsgaard A Hilden J Holm A Brunak S Sensitive quantitative predictions of peptide-MHC binding by a 'Query by Committee' artificial neural network approach Tissue antigens 2003 62 5 378 384 10.1034/j.1399-0039.2003.00112.x 14617044

[B16] Liao WW Arthur JW Predicting peptide binding to Major Histocompatibility Complex molecules Autoimmun Rev 2011 10 8 469 473 10.1016/j.autrev.2011.02.003 21333759

[B17] Feldhahn M Donnes P Thiel P Kohlbacher O FRED--a framework for T-cell epitope detection Bioinformatics 2009 25 20 2758 2759 10.1093/bioinformatics/btp409 19578173 PMC2759545

[B18] Trost B Bickis M Kusalik A Strength in numbers: achieving greater accuracy in MHC-I binding prediction by combining the results from multiple prediction tools Immunome research 2007 3 5 10.1186/1745-7580-3-5 17381846 PMC1847428

[B19] You ZH Lei YK Gui J Huang DS Zhou X Using manifold embedding for assessing and predicting protein interactions from high-throughput experimental data Bioinformatics 2010 26 21 2744 2751 10.1093/bioinformatics/btq510 20817744 PMC3025743

[B20] Karosiene E Lundegaard C Lund O Nielsen M NetMHCcons: a consensus method for the major histocompatibility complex class I predictions Immunogenetics 2012 64 3 177 186 10.1007/s00251-011-0579-8 22009319

[B21] Zhang L Udaka K Mamitsuka H Zhu S Toward more accurate pan-specific MHC-peptide binding prediction: a review of current methods and tools Briefings in bioinformatics 2012 13 3 350 364 10.1093/bib/bbr060 21949215

[B22] Robinson J Mistry K McWilliam H Lopez R Parham P Marsh SG The IMGT/HLA database Nucleic acids research 2011 39 Database D1171 1176 10.1093/nar/gkq998 21071412 PMC3013815

[B23] Singh SP Mishra BN Prediction of MHC binding peptide using Gibbs motif sampler, weight matrix and artificial neural network Bioinformation 2008 3 4 150 155 10.6026/97320630003150 19238237 PMC2639663

[B24] Nielsen M Lund O NN-align. An artificial neural network-based alignment algorithm for MHC class II peptide binding prediction BMC bioinformatics 2009 10 296 10.1186/1471-2105-10-296 19765293 PMC2753847

[B25] Madden DR The three-dimensional structure of peptide-MHC complexes Annual review of immunology 1995 13 587 622 10.1146/annurev.iy.13.040195.003103 7612235

[B26] Miyazawa S Jernigan RL Estimation of effective interresidue contact energies from protein crystal structures: quasi-chemical approximation Macromolecules 1985 18 3 534 552 10.1021/ma00145a039

[B27] Betancourt MR Thirumalai D Pair potentials for protein folding: choice of reference states and sensitivity of predicted native states to variations in the interaction schemes Protein science: a publication of the Protein Society 1999 8 2 361 369 10048329 10.1110/ps.8.2.361 PMC2144252

[B28] Peters B Bui HH Frankild S Nielson M Lundegaard C Kostem E Basch D Lamberth K Harndahl M Fleri W A community resource benchmarking predictions of peptide binding to MHC-I molecules PLoS computational biology 2006 2 6 e65 10.1371/journal.pcbi.0020065 16789818 PMC1475712

[B29] Sette A Sidney J Nine major HLA class I supertypes account for the vast preponderance of HLA-A and-B polymorphism Immunogenetics 1999 50 3 201 212 10.1007/s002510050594 10602880

[B30] Vita R Zarebski L Greenbaum JA Emami H Hoof I Salimi N Damle R Sette A Peters B The immune epitope database 2.0 Nucleic acids research 2010 38 Database D854 862 10.1093/nar/gkp1004 19906713 PMC2808938

[B31] Nielsen M Lundegaard C Blicher T Lamberth K Harndahl M Justesen S Roder G Peters B Sette A Lund O NetMHCpan, a method for quantitative predictions of peptide binding to any HLA-A and-B locus protein of known sequence PloS one 2007 2 8 e796 10.1371/journal.pone.0000796 17726526 PMC1949492

